# Severe Hypoalbuminemia at Admission Is Strongly Associated with Worse Prognosis in Older Adults with SARS-CoV-2 Infection

**DOI:** 10.3390/jcm10215134

**Published:** 2021-10-31

**Authors:** Isabel Arnau-Barrés, Ana Pascual-Dapena, Inmaculada López-Montesinos, Silvia Gómez-Zorrilla, Luisa Sorlí, Marta Herrero, Xavier Nogués, Claudia Navarro-Valls, Beatriz Ibarra, Lizzeth Canchucaja, Elizabeth da Costa Venancio, Fabiola Blasco-Hernando, Juany Cruz, Olga Vázquez, Ramón Miralles, Natalia García-Giralt, Robert Güerri-Fernández

**Affiliations:** 1 Department of Geriatrics, Hospital del Mar, 08003 Barcelona, Spain; iarnau@psmar.cat (I.A.-B.); mherrero@psmar.cat (M.H.); bibarra@psmar.cat (B.I.); lcanchucaja@psmar.cat (L.C.); edacosta@psmar.cat (E.d.C.V.); ovazquez@psmar.cat (O.V.); 2 Departament de Medicina, Universitat Autònoma de Barcelona, 08193 Barcelona, Spain; anapascuald@gmail.com (A.P.-D.); ramon.miralles@uab.cat (R.M.); 3 Facultad de Ciencias de la Salud y de la Vida, Universitat Pompeu Fabra, 08002 Barcelona, Spain; 4 Department of Infectious Diseases, Institute Hospital del Mar of Medical Research (IMIM), Hospital del Mar, 08003 Barcelona, Spain; ilopezmontesinos@psmar.cat (I.L.-M.); sgomezzorrilla@psmar.cat (S.G.-Z.); lsorli@psmar.cat (L.S.); claudia.navarro.valls@psmar.cat (C.N.-V.); fblasco@psmar.cat (F.B.-H.); jcruz@psmar.cat (J.C.); 5 Department of Internal Medicine, Institute Hospital del Mar of Medical Research (IMIM), Hospital del Mar, 08003 Barcelona, Spain; xnogues@psmar.cat (X.N.); ngarcia@imim.es (N.G.-G.)

**Keywords:** SARS-CoV-2, older adults, albumina, outcomes

## Abstract

Serum albumin levels have been associated with prognosis in several conditions among older adults. The aim of this study is to assess the prognostic value in mortality of serum albumin in older adults with SARS-CoV-2 infection. Methods. Cohort observational study with consecutive older-adults (≥65 years old), with confirmed SARS-CoV-2 infection admitted to a university hospital between March–May 2020. A logistic regression model was fitted to assess the impact of albumin levels on in-hospital mortality adjusted by potential confounders. Results. Among a total of 840 patients admitted to the hospital, 405 (48%) were older adults with a total of 92 deaths (23%) among them. Those who died were older, had more comorbidities, higher inflammation status and lower levels of serum albumin at admission [3.10 g/dL (0.51) vs. 3.45 g/dL (0.45); *p* < 0.01. Serum albumin levels at admission were negatively correlated with inflammatory markers such as C-Reactive protein (Pearson Coeff −0.4634; *p* < 0.001) or IL-6 (Pearson’s Coeff −0.244; *p* = 0.006) at admission but also to other clinical outcomes such time to clinical stability (Pearson’s Coeff −0.259; *p* < 0.001). Severe hypoalbuminemia associated with increased risk of mortality was defined as ≤3 g/dL at admission according to the AUC/ROC analysis (0.72 95% CI 0.63–0.81) In a multivariate logistic regression model adjusting by age, inflammation, comorbidities and severity at admission severe hypoalbuminemia was a strong predictor of in-hospital mortality (OR 2.18 95% CI 1.03–4.62; *p* = 0.039). Conclusion. Severe hypoalbuminemia with ≤3 g/dL is an independent risk factor for mortality among older adults with SARS-CoV-2 infection. There is a consistent correlation between albumin levels and inflammatory biomarkers. Further studies are needed to determine whether the supplementation of albumin as coadjuvant treatment will have a positive impact on the prognosis of this infection.

## 1. Introduction

It is well-known that the nutritional status is associated with overall health, and therefore with the morbidity, risk of hospitalization, and mortality [1]. The nutritional status depends on various factors such inadequate diet or inadequate intake, but it seems to be independent of ageing itself [2].

Assessment of the nutritional state may be done by titrating albumin serum levels, which is characterized as a biochemical marker widely used in clinical practice. Albumin has a keystone role as a transporter of numerous endogenous and exogenous compounds, but also maintaining oncotic pressure. In fact, low-serum albumin may precipitate heart failure and is independently associated with increased risk of death [3].

Despite the limitation coming from its prolonged median life that interferes with the detection of acute changes in the nutritional status, serum levels of albumin are strongly related to increases in morbidity (longer hospital stay, poor wound healing) and mortality in subjects with chronic or acute diseases [4]. In the clinical setting, albumin is one of the most frequently used variables to compose prognostic scores, being also considered the best isolated index of prediction of complications, since it has been described as an independent risk factor for mortality [5,6].

Hypoalbuminemia occurs frequently among older adults [7]. Its prevalence ranges from 4% to 50% depending on whether patients live at home or in hospice and whether they are bedridden or not [8]. In older adults, hypoalbuminemia can be a physiological finding, since with ageing, the serum albumin level decreases, reducing the albumin level by 20% in individuals over 70 years of age [7,9]. This reduction over the years may impact negatively in the response against injury which is reflected in longer hospitalization time and increases in morbidity [10]. Therefore, the role of albumin is far beyond as a nutritional biomarker, and may contribute to enhanced response against injuries such as SARS-CoV-2 infection [11,12].

During the last months, SARS-CoV-2 infection has especially affected the older adult population. COVID-19 may be an ideal scenario to confirm the role of albumin as an intermediate factor, and as an independent factor of severity and poorer prognosis. The first waves of the pandemic have hit this population deeply and the mortality and comorbidity related to the infection have been strong. Many prognostic factors have raised from interleukin-6 (IL-6) to C-reactive protein or ferritin [13]. In addition, serum albumin levels have been proposed as a biomarker that represents the prior status of the individual, as a prognostic factor and also as a result of the acute response to infection [14,15]. Moreover, its role is far beyond a simple biomarker, but a potential physiological factor that leads to the response against the infection. There is scarce data on the adverse outcomes that may occur in older adults who have reduced serum albumin during acute infections. However, there is increasing evidence that shows that might be related.

The aim of this study is to assess the prognostic utility of serum albumin among older adults admitted for SARS-CoV-2 infection in terms of all-cause mortality and also to identify the predictors of hypoalbuminemia, such as its association with inflammation. Findings from this study may bring to light new areas of study that may improve outcomes among older adults with acute severe infection.

## 2. Patients and Methods

We performed a retrospective analysis of all individuals aged ≥65 years old that had been prospectively recorded after admission at Hospital del Mar in Barcelona, Spain. During the COVID-19 crisis the hospital was adapted to the SARS-CoV-2 infection care by revising and homogenizing protocols with a unique electronic medical record and a centralized registry of all individuals admitted to the COVID-19 unit. For this study, we included all patients admitted to the COVID-19 unit for ≥48 h between March and May, 2020. Older adults were defined as individuals ≥65 years. Admitted individuals had a complete per-practice protocol blood test that included albumin, kidney and liver function, blood count cell, C-reactive protein, Fibrinogen, and D-dimer on the day of admission. A complete clinical history was compiled including clinical diagnostic criteria (respiratory symptoms such as dyspnea, cough, sore throat, changes in taste/smell; or uni-/bilateral interstitial infiltrates in chest X-Ray), symptoms evolution, and treatments. Admission criteria to the unit was having a confirmed SARS-CoV-2 infection by PCR.

Clinical variables, data source, and study outcome.

Data was obtained from electronic clinical records. Laboratory results were extracted using standardized data collection. Clinical severity was assessed at admission with MEWS score [16,17]. Comorbidity was assessed using the Charlson Comorbidity Index [18] and categorized into no comorbidities, mild (1–2 comorbidities), or severe (≥3 comorbidities). Severe hypoalbuminemia was defined as serum albumin value ≤3 g/dL after a receiver operating characteristic (ROC) cutoff point for mortality was obtained.

Key outcomes included time to clinical stability (defined as the time elapsed since the patient’s admission to: oxygen saturation >94% (FiO_2_ 21%), normalized level of consciousness (baseline), HR <100 rpm, systolic BP >90mm Hg, Temperature <37.2 °C), or in-hospital mortality.

### 2.1. Ethics Considerations

The Institutional Ethics Committee of Hospital del Mar of Barcelona approved the study and due to the nature of the retrospective data review, waived the need for informed consent from individual patients (CEIm 2020/9352).

### 2.2. Statistical Analysis

Continuous variables are expressed as means and Standard Deviation (SD). Categorical variables are expressed as frequencies (percentages). Continuous variables were compared using the Student *t*-test or Mann–Whitney U test, and categorical variables using χ^2^ test or the Fisher exact test, as required. Appropriate coefficient tests were used for correlation among various continuous variables.

A multivariate logistic regression analysis was fitted to assess the effect of severe hypoalbuminemia to predict mortality. Co-variables included in the model were the previously known risk factors according to the literature and variables with differences found between survival and non-survival group after study of collinearity and interactions. When differences in two variables measuring the same output such C-Reactive Protein or IL-6 the most significant was retained in the model. The final multivariate model was fitted using a backward stepwise approach, retaining only those variables with a *p*-value < 0.10. Cox proportional hazards model was used to estimate hazard ratios (HR) according to the albumin level (severe vs. non-severe hypoalbuminemia) the risk of in-hospital death. Proportional hazard assumption was verified in each model. Patient discharge was considered as competitive event. Therefore, the Fine and Gray model (sensitivity analyses accounting for a competing risk) was fitted to estimate sub-distribution hazard ratios (SHR) of the outcome. HR and SHR are reported with 95% confidence intervals (95% CI).

The level of significance in this study was set at a *p* ≤ 0.05 and confidence interval of 95%. All statistical analyses were performed using STATA/MP V.14.0.

## 3. Results

Among the 840 patients admitted during the period of observation, 405 (48%) were older adults. Of the 405 individuals included, 92 patients (23%) died during the hospitalization. The main baseline characteristics of patients stratified according to survivors vs. non-survivors are shown in Table 1. There were significant differences between both groups where the non-survivors were older (*p* < 0.001) and had more cardiovascular disease (*p* = 0.008) and in the initial presentation the non-survival group showed higher inflammation parameters than survivors as measured by C-Reactive protein [14.1 (10.9) vs. 9.1 mg/dL (16.1); *p* = 0.017, respectively] and by IL-6 [183 (220) vs. 76.9 pg/mL (130); *p* = 0.001, respectively]. Besides, non-survivors had significant lower levels of serum albumin compared to survivors at admission [3.10 g/dL (0.51) vs. 3.45 g/dL (0.45); *p* < 0.01, respectively].

### 3.1. Subsection

#### 3.1.1. Older Adults with and without Severe Hypoalbuminemia

**Figure 1:**
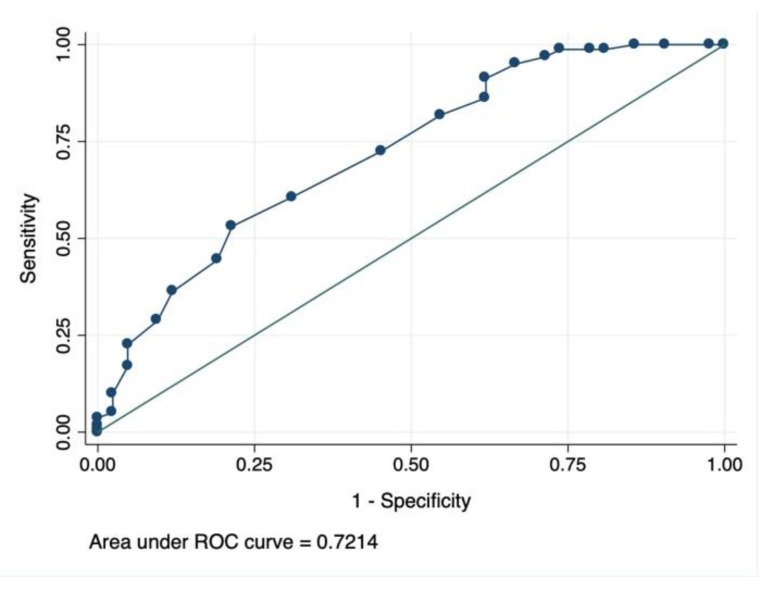
Area under ROC curve for the outcome of mortality with albumin.

Serum albumin levels at admission were found to be different among groups and therefore, they might be considered a relevant prognostic factor for in-hospital mortality. ROC curve analysis was performed to determine the value of serum albumin for predicting mortality during the in-hospital stay. As shown in Figure 1 the area under the ROC curve (AUC) for the outcome of mortality was 0.72 (95% CI 0.63–0.81) for a serum albumin ≤3 g/dL. According to this ROC cutoff for mortality, severe hypoalbuminemia was defined as a serum albumin ≤3 g/dL.

A significantly higher proportion of patients in the non-survivors group had admission serum albumin levels of ≤3 g/dL than those that survived [11 (13%) vs. 14 (6%); *p* = 0.035, respectively].

Individuals with severe hypoalbuminemia presented a more severe disease measured by MEWS score [2 (2–3) in sever hypoalbuminemia group vs. 1 (1–2) in those with albumin >3g/dL; *p* < 0.001]. Remarkably, older adults with severe hypoalbuminemia had significantly higher mortality rates than those without hypoalbuminemia [84 (21%) vs. 11 (44%); *p* < 0.001, respectively]. There were no differences in terms of age or comorbidities associated with higher mortality in SARS-CoV-2 infection such high blood pressure, diabetes or chronic kidney disease between these groups (Table 2). Nevertheless, globally, individuals with severe hypoalbuminemia were more comorbid when scored by Charlson comorbidity index (Table 2).

#### 3.1.2. Correlation of Albumin and Clinical Markers

Serum albumin levels at admission were negatively correlated with inflammatory markers such as C-Reactive protein at admission (Pearson Coeff −0.4634; *p* < 0.001) or IL-6 at admission (Pearson’s Coeff −0.244; *p* = 0.006) but also to other clinical outcomes such time to clinical stability (Pearson’s Coeff −0.259; *p* < 0.001).

We found no correlation between albumin levels at admission and age (Pearson’s coeff −0.071; *p* = 0.372). Cox analysis showed a survival reduction in individuals with severe hypoalbuminemia compared to individuals with non-severe hypoalbuminemia (HR: 2.27 [95 CI%: 1.43–3.61]) that was confirmed after competing risk analysis (SHR: 1.71 [95 CI%: 1.22–1.44]).

In a multivariate logistic regression model, severe hypoalbuminemia increased the risk of in-hospital mortality (OR 2.18 95% CI 1.03–4.62; *p* = 0.039) after adjusting by age, gender, inflammation markers (C-Reactive protein), comorbidities and severity of the episode measured by MEWS score (Table 3).

## 4. Discussion

We report a significant increase in risk of mortality among older adults with severe hypoalbuminemia defined as serum albumin level less than ≤3 g/dL at the time of admission for a SARS-CoV-2 infection.

Albumin has previously been described as an independent factor for mortality in other conditions such as cardiovascular disease, osteoporotic hip fracture, stroke and also sepsis [4,19,20,21,22]. Our series with SARS-CoV-2 infected patients as a model of severe infection confirms these previous results and we found that albumin levels correlate with in-hospital mortality, and especially severe low levels stablished at ≤3 g/dL.

The importance of albumin in these processes remains to be elucidated, since low albumin levels might be acting as a surrogate of the functional reserve or fragility of the older people along with the physical activity and the nutritional status. Therefore, an individual with lower albumin levels would be less prepared to respond against infection, leading to an exacerbation of the disease and an increased mortality.

Otherwise, in a critical illness such severe SARS-CoV-2 infection, hypoalbuminemia might also be the consequence of the severe infection itself through a variety of mechanisms. On one side, the increased systemic inflammation with mediators such IL-6 or TNF alpha led to capillary leakage increasing distribution of albumin to extravascular compartment, reducing intravascular oncotic pressure and consequently increasing the tissue edema. This might increase the risk of non-cardiogenic pulmonary edema [6,23,24], one of the heralds of SARS-CoV-2 infection [25]. This distribution of the albumin from the intravascular to the extravascular compartment could explain the rapid reduction of albumin levels observed in some critical patients [8,26]. Although, there might be both an increased rate of clearance and a diminished rate of synthesis by the liver as a consequence of the severe inflammation (1). It is highly unlikely that hypoalbuminemia during acute infection might be related with reduced synthesis, due to the long half-life of albumin. Indeed, it is observed in liver cirrhosis, as a chronic condition, while in acute hepatitis hypoalbuminemia is seldom observed [8,27]. This leads us to entertain the hypothesis that increased catabolism of albumin is induced by infection related inflammation.

In our series, we found a significant and consistent negative correlation between inflammatory markers at admission and albumin levels adding more weight to the hypothesis that, regardless of prior status, the intensity of SARS-CoV-2-induced inflammation would impact albumin levels, and consequently disease prognosis. It could be plausible that the severity of the infection is, at the end, responsible for a greater loss in albumin. Low albumin at admission could thereby be reflecting a more severe disease, with increased viral replication and higher inflammation state driving at the end to worse prognosis.

We report that individuals with severe hypoalbuminemia were not different in terms of age, or even the main risk mortality factors described for SARS-CoV-2, such as high blood pressure or diabetes [28]. However, higher Charlson comorbidity score and more dementia were detected in the severe hypoalbuminemia group. Interestingly, the association between severe hypoalbuminemia and in-hospital mortality remained significant after adjusting by age, inflammatory status, comorbidities and severity. According to our results, the concurrence of the inflammatory status derived from SARS-CoV-2 infection in older adults in addition to the less functional reserve in older adults might contribute to the hypoalbuminemia leading to clinical manifestations of the infection itself.

A limitation of the study is that we do not have the prior albumin levels of the individuals before SARS-CoV-2 infection, nor their performance or nutritional status. Hence, we cannot know whether hypoalbuminemia was present before the infection or is a consequence. We hypothesize that the reduction in albumin levels was induced by the infection due to the aforementioned mechanisms (increased clearance, reduced synthesis and distribution to other compartments) through inflammation, leading to physiological changes due to hypoalbuminemia in the course of the disease. The consequence is that severe hypoalbuminemia at admission significantly increased the risk of in-hospital mortality. Whether a reposition of albumin could improve the prognosis of these selected group of individuals remains to be clarified and requires further investigations.

Although the analysis of albumin as a prognostic factor has already assessed in SARS-CoV-2 infections (14, 15), our study has some differences with prior studies and some strengths that must be taken into account. First, the sample size of our study is bigger than previous ones, and the results observed are in the same line as prior reports increasing evidence of the value of serum albumin as a prognostic factor, and also as a potential therapeutic target. Secondly, although this is a single center study, it was conducted during the first wave of the SARS-CoV-2 pandemic. This study is focused on an older adult population. This group is usually underrepresented in other series and deserves a specific analysis due to the different behavior of older adults as a group. Our hospital was the drainage of several facilities and centralized the care of older patients with the most severe infection. In fact, our study comprised a representative sample of an older adult population, belonging to a large city with high incidence of SARS-CoV-2 infection, which makes the results directly relevant to the clinical practice. And finally, we report a cut-off of albumin level at 3 g/dL where the risk of death is greater and were the early therapeutic intervention might have a significant impact.

## 5. Conclusions

We can conclude that severe hypoalbuminemia established in our study at ≤3 g/dL is an independent risk factor for mortality among older adults with SARS-CoV-2 infection. In addition, albumin levels impacted the time to clinical recovery of the individuals. The consistent association between inflammation and albumin makes the hypothesis of an inflammatory result as responsible for albumin reduction translating into a more severe infection. Consequently, individuals with worse functional reserve and higher inflammation were the ones more impacted by low albumin levels and worse prognosis. Further studies are required to determine the role of albumin supplementation as coadjutant treatment in a such critical infection.

## Figures and Tables

**Table 1:** Baseline characteristics among older adults with SARS-CoV-2 infection stratified according to in-hospital mortality.

	Overall (*n* = 405)	Survivors (*n* = 313)		Non-Survivors (*n* = 92)		*p*-Value
Age, years	79 (8.6)	78	(8.6)	83	(8.4)	**<0.001**
Male (%)	180 (46%)	122	(44%)	46	(50%)	0.432
Comorbidities						
Current smoker (%)	11 (3%)	9	(3%)	2	(2%)	0.702
High blood pressure (%)	275 (72%)	202	(64%)	73	(78%)	**0.013**
Diabetes Mellitus (%)	106 (28%)	83	(26%)	23	(24%)	0.719
Chronic lung disease (%)	40 (10%)	29	(9%)	11	(12%)	0.472
Chronic heart disease (%)	86 (21%)	57	(18%)	29	(31%)	**0.008**
Chronic renal disease (%)	158 (39%)	129	(41%)	29	(31%)	0.078
Chronic liver disease (%)	21 (5%)	15	(5%)	6	(6%)	0.530
Charlson Comorbidy Index, median (IQR)	2 (1–3)	1	(0–3)	2	(1–5)	**<0.001**
No comorbitiy, *n* (%)	142 (35%)	122	(39%)	20	(22%)	**0.002**
Medium-low (1–2), *n* (%)	124 (30%)	97	(31%)	27	(29%)	0.535
High (≥3), *n* (%)	139 (34%)	93	(29%)	46	(48%)	**0.001**
Clinical Markers at Admission						
C-Reactive Protein mg/dL	11.1 (14.7)	9.1	(16.1)	14.1	(10.9)	**0.017**
Procalcitonin mg/dL	0.83 (2.7)	0.68	(2.6)	1.16	(3.1)	0.322
IL-6 pg/mL	94.2 (154)	76.9	(130.2)	183.3	(220.1)	**0.001**
Albumin mg/dL	3.45 (0.45)	3.52	(0.43)	3.10	(0.51)	**<0.001**
D-Dimer UI/L	2786 (5658)	2602	(5897)	3161	(4118)	0.488
Creatinin mg/dL	1.22 (0.86)	1.06	(0.62)	1.64	(1.26)	**<0.001**
PaFi	205 (108)	228	(104)	137	(85)	**<0.001**
Median MEWS (IQR)	2 (1–3)	1	(1–2)	2	(2–3)	**<0.001**

Data are presented as mean and standar devation unless otherwise specified. IQR (Interquartile Range) MEWS (Modified Early Warning Score) PaFi (PaO_2_/FiO_2_ ratio) IL-6 (Interleukin-6).

**Table 2:** Clinical differences between older adults with SARS-CoV-2 infection with serum albumin at admission >3 g/dL vs. ≤3 g/dL.

	Albumin > 3 g/dL (*n* = 380)		Albumin ≤ 3 g/dL (*n* = 25)		*p*-Value
Age, years	79	(8.6)	80	(8.4)	0.543
Male sex (%)	120	(42%)	14	(56%)	0.194
Comorbidities					
Current smoker (%)	11	(4%)	0	(0%)	0.320
Hypertension (%)	209	(72%)	21	(84%)	0.214
Diabetes Mellitus (%)	77	(26%)	10	(40%)	0.156
Chronic lung disease (%)	33	(6%)	11	(12%)	**0.027**
Chronic heart disease (%)	52	(22%)	26	(31%)	0.098
Chronic renal disease (%)	63	(27%)	24	(29%)	0.787
Chronic liver disease (%)	12	(5%)	5	(6%)	0.757
Charlson Comorbidy Index, median (IQR)	1	(0-3)	2	(1–5)	**<0.001**
No comorbitiy, *n* (%)	70	(30%)	16	(19%)	0.060
Medium-low (1–2), *n* (%)	82	(35%)	26	(31%)	0.535
High (≥3), *n* (%)	79	(34%)	40	(48%)	**0.019**
Clinical Markers at Admission					
C-Reactive Protein mg/dL	10.1	(16.1)	14.1	(10.9)	**0.047**
Procalcitonin mg/dL	0.68	(2.6)	1.16	(3.1)	0.322
IL-6 pg/mL	76.9	(130.2)	183.3	(220.1)	**0.001**
D-Dimer UI/L	2642	(5425)	4585	(7118)	0.138
Creatinin mg/dL	1.19	(0.82)	1.54	(1.16)	**0.032**
PaFi	210	(104)	159	(116)	0.072
Median MEWS (IQR)	1	(1–2)	2	(2–3)	**<0.001**
Time to clinical stability, days	14	(16)	24	(16)	**0.003**
In-hospital mortality, *n* (%)	82	(21)	11	(44)	**0.010**

Data are presented as mean and standard deviation unless otherwise specified. IQR (Interquartile Range). MEWS (Modified Early Warning Score) PaFi (PaO_2_/FiO_2_ ratio) IL-6 (Interleukin-6).

**Table 3:** Adjusted Odds ratios (OR) for in-hospital mortality in older adults admitted with SARS-CoV-2 infection.

	OR Adjusted (95% CI)	*p*-Value
Albumin < 3 g/dL	2.18 (1.03–4.62)	0.039
Age	1.11 (1.09–1.14)	0.047
Male gender	1.03 (0.65–1.63)	0.148
Hypertension	2.38 (1.42–3.99)	0.002
Diabetes mellitus	0.61 (0.33–1.11)	0.103
Chronic heart disease	1.64 (0.91–2.97)	0.322
Chronic Kidney Failure	0.88 (0.33–1.54)	0.655
High Charlson Comorbidity Index (≥3)	1.28 (1.28–3.52)	0.004
C-Reactive Protein > 5 mg/dL	2.03 (1.25–3.30)	0.007
MEWS score > 3	2.11 (1.28–3.77)	0.003

## Data Availability

We have not planned to upload our data for sharing. This data come from a general database that is being collected in real time information about all the admissions with SARS-CoV-2 infection in the hospital. However, datasets are available from the corresponding author upon reasonable request.
